# Reinforcement of suture lines with aortic eversion in aortic replacement

**DOI:** 10.5830/CVJA-2017-008

**Published:** 2018

**Authors:** Kaya Erhan

**Affiliations:** Private Pendik Regional Hospital, Department of Cardiovascular Surgery, Istanbul, Turkey

**Keywords:** suture technique, ascending aortic aneurysms, dissection, pledgetted

## Abstract

**Background:**

In this study, we describe the technique of eversion of the native aortic tissue to prevent suture line complications, and report on our results with this technique.

**Methods:**

A total of 42 patients who were operated on due to aortic aneurysm were retrospectively assessed. In all patients, an aortic segment of approximately 2 cm, which was left both distally and proximally, was everted to form a doublelayer lumen and the grafts were anastomosed. Postoperative outcomes and long-term follow-up results were assessed.

**Results:**

Aortic root replacement was done in 14 cases and eight subjects underwent concurrent coronary artery bypass surgery. Postoperatively, the average volume of the drainage was 375 ± 75 ml, and there were no re-operations. Twentyseven patients required blood transfusion.

**Conclusion:**

Reinforcement of the anastomosis line via eversion of the native aortic tissue reduced peri-operative blood loss and pseudo-aneurysm and infection, with the advantage of using viable tissue.

## Background

Bleeding at the suture line may be severe enough to necessitate re-do cardiopulmonary bypass in patients undergoing surgical prosthetic graft replacement due to aortic dissection or aneurysm. Various techniques have been reported in an effort to prevent this complication, including the use of pledgetted stitches and/ or bands during anastomosis, placement of additional sutures, use of interrupted pledgetted sutures in the posterior region, use of pledgetted sutures together with aortic inversion, use of bands, inclusion of the graft within the graft, or the use of tissue fibrinogen activators after anastomosis.^1^-^4^

The inflammatory response to foreign pledgetted material or adhesions associated with the use of fibrin tissue adhesives may increase the risk of infection in the long term or may complicate dissection when re-operation is necessary. On the other hand, external eversion of the aortic tissue at the site of anastomosis to obtain a double-layered lumen to reinforce the suture line may offer an alternative to pledgetted sutures or bands, allowing minimal use of foreign material, preservation of tissue viability at the suture line, and reducing the early risk of bleeding and long-term risk of infection.

In this study, we present our results of a group of patients who underwent ascending aortic tube graft replacement with eversion of the aortic tissue in the stump and minimal or no use of pledgetted sutures/bands to avoid postoperative bleeding, pseudo-aneurysm and infection.

## Methods

Patients undergoing surgery due to ascending aortic aneurysm between 1 May 2014 and 31 December 2015 in our unit were included in this retrospective study. Forty-two patients with a diagnosis of ascending aortic aneurysm underwent surgery in this period.

During surgery, aortic tissue was everted without the use of pledgetted sutures or bands in all patients undergoing distal anastomosis, as well as in all patients undergoing proximal anastomosis with tube graft interposition only. In those undergoing aortic root surgery, aortic tissue was everted on the non-coronary site of the proximal anastomosis, while Teflon band reinforcement was done on the right and left coronary sides in those subjects lacking adequate tissue for eversion.

All procedures were performed under general anaesthesia and cardiopulmonary bypass with a median sternotomy. The right axillary artery was used for cannulation in all patients. During surgery, arterial cannulation was performed through the right axillary artery in all patients, while antegrade cerebral perfusion and the open-anastomosis technique were used during distal anastomosis. Except for one patient who had mitral valve repair with bicaval venous cannulation, and another who underwent atrial septal defect (ASD) closure, venous cannulation was performed with a single venous cannula from the right atrium in all patients.

Left heart decompression was achieved via the right superior pulmonary vein and left atrial vent. After cross-clamping at the distal ascending aorta, cardiac arrest was achieved with blood cardioplegia through the aorta in patients with aortic sufficiency, while in those with aortic valve insufficiency, initial cardioplegia was achieved with the retrograde coronary sinus route, followed by selective coronary ostia after aortotomy. In all patients, myocardial protection was maintained continuously via the coronary sinuses after antegrade cardioplegia.

After opening the aneurysmal sac, it was transected proximally and distally, while care was taken to leave approximately 2 cm of aortic tissue, allowing eversion at both ends. Similarly, in patients undergoing coronary re-implantation, aortic tissue adequate for eversion was left intact proximally on the non-coronary side. In our unit, we perform re-implantation of the coronary ostia by leaving a wide margin of aortic tissue around the coronary ostia, using the eversion technique. A proximal anastomosis was then performed by placing the sutures first through the double- layered aortic tissue, then through the graft, using continuous 4/0 prolene sutures without pledgetted sutures or band. In those undergoing aortic root replacements, adequate native aortic tissue was left in the non-coronary sinus area, allowing eversion, while the anastomosis in the right and left coronary sinus area was performed with reinforcement from a Teflon band, since there was insufficient aortic tissue to allow for eversion.

**Fig. 1:**
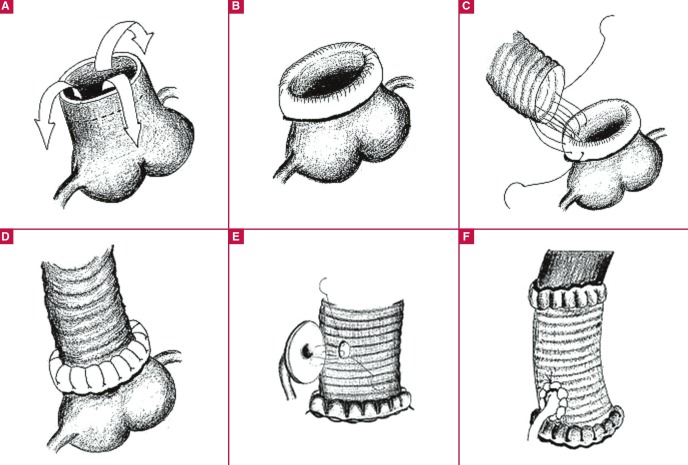
A: We left 2 cm of aortic tissue to allow for eversion of the aorta. B: Double-layered aortic tissue is prepared by everting and suturing 2 cm of aortic tissue. C: Proximal anastomosis is performed using continuous 4/0 prolene sutures. D: View of the ascending aorta after proximal anastomosis. E: In the aortic root replacement, double-layered aortic tissue is prepared at the coronary buttons. F: View of the aorta after coronary anastomosis.)

**Fig. 2:**
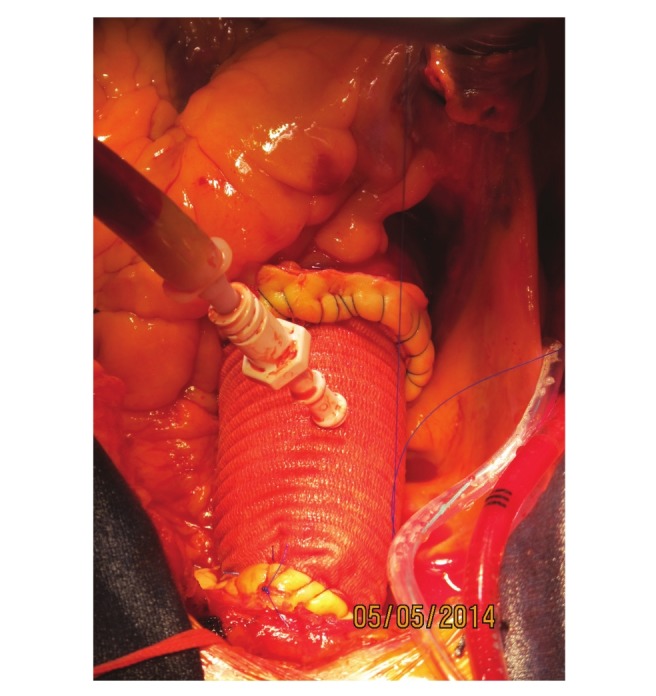
Control of bleeding with administration of cardioplegia via a needle over the graft.

After proximal aortic anastomosis, the coronary arteries were anastomosed to the graft by eversion of the excess aortic tissue in the button (Fig. 1). Subsequently, cardioplegia was administered through a needle over the graft to check bleeding at the proximal anastomosis line and the coronary implantation suture lines (Fig. 2). A clamp was then placed on the innominate artery and the cross-clamp was removed. The aortic tissue was everted to accomplish the distal anastomosis of the graft under antegrade cerebral perfusion and mild hypothermia.

In patients with additional cardiac pathologies, aortic replacement was completed after the surgical procedure for the cardiac pathology had been carried out. In patients undergoing aortic root replacement with a valved conduit, a modified Bentall procedure with flanged graft was used, as we believe that this approach may help reduce the risk of tissue–prosthesis incompatibility as well as the risk of bleeding, in addition to shortening the duration of anastomosis.^5^ When simultaneous coronary bypass surgery was done, proximal anastomoses were .performed during the warming phase after ascending aortic tube graft implantation. After cardiopulmonary bypass (CPB) was terminated, the surgery was completed with haemostatic control.

## Statistical analysis

Statistical assessments were performed using Microsoft Excel software. All numerical data are presented as mean ± standard error, while categorical variables are presented as percentages.

## Results

The mean age of the patient group was 58 ± 2 years and 54.8% of the study population was male. Demographic characteristics and pre-operative data of the study patients are shown in Table 1.

**Table 1:** Pre-operative demographical data of the patients

*Value*	*Variable*
Age (mean ± SD))	58.2 ± 13.9
Male, n (%)	23 (54.8)
Hypertension, n (%)	21 (50)
Diabetes, n (%)	1 (2.3)
Chronic renal failure, n (%)	0 (0)
COPD, n (%)	6 (14.2)
Coronary artery disease, n (%)	8 (19)
History of CVA, n (%)	2 (4.8)
Re-operation, n (%)	1 (2.3)
Pre-operative EF (%) (mean ± SD)	58.2 ± 13.8
Aortic insufficiency, n (%)	22 (52.4)

SD: standard deviation, EF: ejection fraction, COPD: chronic obstructive pulmonary disease, CVA: cerebrovascular accident.

Aortic root replacement was performed in 14 patients. Four received surgical valvular repair, and a total of 18 patients underwent aortic valve replacement, including a modified Bentall procedure with flanged grafts in 10 patients (Table 2).

**Table 2:** Surgical procedures

*Variable*	*Value*
Ascending aortic repair without valve procedure	20
Ascending aortic repair with valve procedure	22
Bentall procedure	10
David operation	4
Separated graft interposition	8
Concomitant surgical intervention	
AVR	18
CABG	8
Mitral valve repair	1
ASD repair	1

AVR: aortic valve replacement, CABG: coronary artery bypass grafting, ASD: atrial septal defect.

The mean CPB and cross-clamp times were 80 ± 18 and 53 ± 18 minutes, respectively. The mean postoperative drainage volume was 375 ± 75 ml. The mean transfusion rate of erythrocyte suspension was 1.1 ± 0.3 units (Table 3). No patient required revision surgery and the average duration of hospital stay was 7.9 ± 1.4 days.

**Table 3:** Surgical findings

*Variable*	*Value*
Duration of cross clamp, min (mean ± SD)	52.9 ± 17.7
Duration of cardiopulmonary bypass, min (mean ± SD)	79.8 ± 18.5
Drainage, ml (mean ± SD)	375 ± 75
Revision, n	0
Erythrocyte replacement, units (mean ± SD)	1.1 ± 0.3
Duration of intubation, hours (mean ± SD)	5.3 ± 1.1
Postoperative EF, % (mean ± SD)	54.8 ± 6.3
Duration of hospitalisation, days (mean ± SD)	7.9 ± 1.4

SD: standard deviation, EF: ejection fraction.

## Discussion

Among cardiac operations, aortic surgery is generally associated with higher volumes of blood loss due to a number of factors, including thinned, atherosclerotic, calcific or fragile aortic tissue, and also due to re-implantation of the coronary arteries. Following an anastomosis, bleeding occurring posteriorly poses a particular challenge since it may require re-initiation of CPB.

Different methods have been reported to reduce the risk of postoperative bleeding in these patients.^1^-^4^ In our practice, autogenous aortic tissue is generally used to reduce the risk of bleeding based on the advantage of tissue continuity. Also, easier control of bleeding with additional sutures on the native aortic tissue represents an additional benefit of this approach. We therefore perform anastomosis after obtaining double-layered aortic tissue with eversion of the autogenous aorta.

Among our 42 patients undergoing ascending aorta replacement using this technique, no complications occurred and there were no cases requiring re-operation. The average drainage volume was 375 ml. No cases of postoperative morbidity/ mortality associated with pseudo-aneurysms, complications due to the use of foreign materials, or bleeding were recorded.

Prolonged CPB, hypothermia and administration of heparin are associated with an increased risk of postoperative bleeding in patients undergoing cardiac surgery,^6^ leading to increased requirement for transfusion, with a subsequent increase in the risk of infection, anaphylaxis and renal/pulmonary injury.^6^ With this technique as described above, an average of 1.1 ± 0.3 units of erythrocyte suspension were transfused in approximately two-thirds of our patients.

Pseudo-aneurysms may arise at the suture line after ascending aorta graft replacement, or infections may cause dehiscence at the suture line,^7^-^10^ elevating the risk of mortality and need for re-operation. Higuchi et al.^11^ reported lower risk of bleeding using continuous sutures for anastomosis after the inclusion of a 7-cm segment of Dacron tube graft, folded with three sutures to achieve a double-layered structure. However, this approach may be expected to increase the early risk of thrombosis formation in the graft as well as embolic risk, since the contact surface between the synthetic graft material and the aorta is increased.

On the other hand, the method described above, involving eversion of the autogenous aortic tissue would not only reduce the amount of intra-luminal tissue, but would also exploit the advantage of using autogenous tissue. Use of as much viable tissue as possible during graft replacement may also hasten the postoperative healing process at the suture lines, reducing the risk of pseudo-aneurysm.

Ohata et al.^3^ reported the use of a graft interposition technique in which the aortic tissue is folded inside, leaving a felt band in the outer layer. In this technique, continuous prolene sutures were preferred, and in contrast with our approach, the graft was approximated to the aorta using four mattress sutures.

Use of Surgicel to prevent bleeding at the suture line during surgery may lead to a pressure effect on the coronary artery, while tissue adhesives may compress coronary ostia from the outer surface and lead to ischaemia, embolism, necrosis of the aortic tissue and prosthetic valve dysfunction.^3^,^12^ Wrapping with bovine pericardium has also been proposed for bleeding control at the suture lines.^12^ Graft infection in the ‘dead space’ has been reported, even with wrapping using autogenous aortic tissue in ascending aortic grafting.^13^ The infection risk due to the formation of a potential dead space between the two grafts, as well as the degree of inflammation caused by the wrapping of a synthetic graft using a second biological graft are currently unknown.

When there is no adequate space for cross-clamping in ascending aortic lesions, antegrade perfusion with axillary artery cannulation may be reliably used.^14^ In order to achieve better exposure of the distal anastomosis and to perform aortic tissue eversion, we prefer an open anastomosis technique for the distal anastomosis, using selective cerebral perfusion via the axillary artery in all cases. Using this approach, there were no postoperative complications.

This study has the obvious limitations of retrospective studies. All data were obtained from medical records. Since we routinely perform reinforcement of suture lines with aortic eversion in ascending aortic surgery, there was no control group. Prospective, randomised studies are needed to improve our results.

## Conclusion

In ascending aortic surgery, the thin, fragile aorta is subjected to eversion to obtain a double-layered tissue. In this technique using viable aortic tissue, the risk of bleeding, pseudo-aneurysms and dehiscence are reduced. The ascending aortic anastomosis technique with aortic eversion is a simple procedure that may be reliably preferred in aortic surgery, with reduced postoperative complication rates.
